# The Effect of Strontium Ranelate on Fracture Healing: An Animal Study

**DOI:** 10.1155/2020/1085324

**Published:** 2020-11-24

**Authors:** Ourania I. Koukou, Lampros D. Pappas, Pelagia Chloropoulou, Maria A. Kouroupi, Konstantinos I. Koukos, Georgia Karpathiou, Antonios A. Galanos, Georgios I. Drosos, Evaggelos Magnisalis, Alexandra N. Giatromanolaki, Dionysios Alexandros Verettas

**Affiliations:** ^1^Private Practice, Komotini 69132, Greece; ^2^Department of Orthopaedics, General Hospital of Didymoteicho, Didymoteicho 68300, Greece; ^3^Department of Anesthesiology, School of Medicine, Democritus University of Thrace, Alexandroupolis 68100, Greece; ^4^Department of Pathology, School of Medicine, Democritus University of Thrace, Alexandroupolis 68100, Greece; ^5^Department of Orthopaedics, General Hospital of Preveza, 48100, Greece; ^6^Department of Pathology, University Hospital of Saint-Etienne, 42055 CEDEX2 Saint-Etienne, France; ^7^Laboratory of Research of the Musculoskeletal System, School of Medicine University of Athens, 14561, Greece; ^8^Department of Orthopaedics, School of Medicine, Democritus University of Thrace, Alexandroupolis 68100, Greece; ^9^Laboratory of Bioengineering, BioHexagon Ltd, Athens 17124, Greece; ^10^Democritus University of Thrace, Alexandroupolis 68100, Greece

## Abstract

**Background:**

*S*trontium ranelate (StR) is an antiosteoporotic agent previously utilized for the enhancement of fracture union. We investigated the effects of StR on fracture healing using a rabbit model.

**Methods:**

Forty adult female rabbits were included in the study and were divided in 2 equal groups, according to StR treatment or untreated controls. All animals were subjected to osteotomy of the ulna, while the contralateral ulna remained intact and served as a control for the biomechanical assessment of fracture healing. Animals in the study group received 600 mg/kg/day of StR orally. All animals received ordinary food. At 2 and 4 weeks, all animals were euthanatized and the osteotomy sites were evaluated for healing through radiological, biomechanical, and histopathological studies.

**Results:**

The treatment group presented statistically significant higher callus diameter, total callus area, percentage of fibrous tissue (*p* < 0.001), vessels/mm^2^, number of total vessels, and lower osteoclast number/mm^2^ (*p* < 0.05) than the control group at 2 weeks. Additionally, the treatment group presented significantly higher percentages of new trabecular bone, vessels/mm^2^, osteoclast number/mm^2^, and lower values for callus diameter, as well as total callus area (*p* < 0.05), than the control group at 4 weeks. At 4 weeks, in the treatment group, force applied (*p* = 0.003), energy at failure (*p* = 0.004), and load at failure (*p* = 0.003) were all significantly higher in the forearm specimens with the osteotomized ulnae compared to those without. Radiological bone union was demonstrated for animals receiving StR at 4 weeks compared with controls (*p* = 0.045).

**Conclusion:**

StR appears to enhance fracture healing but further studies are warranted in order to better elucidate the mechanisms and benefits of StR treatment.

## 1. Introduction

Fracture healing is a complex biological and biomechanical process, and the successful completion of which significantly reduces the economic burden of healthcare on fracture treatment [1, 2]. The healing potential depends on several factors, such as displacement of the fracture ends, vascularity and hematoma formation, method of stabilization, patient's age, and presence of comorbidities, including diabetes, tobacco smoking, and poor dietary habits [3]. Approximately 5–10% of bone fractures lead to nonunion and incomplete healing [4].

Fracture healing complications are associated with considerable healthcare resource expenditure, functional impairment, and increased use of opioid medications [5]. Thus, enhancement of fracture healing is an important parameter of the management of bone fractures. Over the years, there is an increasing interest regarding the effect of agents used in the treatment of osteoporosis in the fracture healing process [6].

Strontium ranelate (StR) is composed of two atoms of stable strontium (Sr^+2^) and the ranelic acid organic molecule [7]. It is an extensively studied drug, mainly used in the treatment of osteoporosis [8]. Unlike other antiosteoporosis compounds, StR presents a dual effect on bone metabolism, inhibiting bone resorption, and maintaining bone formation [9]. In particular, in vitro studies have shown that StR inhibits osteoclast activity and stimulates osteoblast proliferation and collagen synthesis [9]. Furthermore, it has been shown to increase the expression and protein levels of osteoprotegerin as well as reducing the expression and protein levels of RANKL [10, 11]. In cultured osteoblasts, StR increased osteoblast formation and expression of mature osteoblast markers in primary osteoblasts and promoted bone nodule formation [9]. In experimental animal studies, long-term StR treatment has been shown to increase bone strength and bone mass and reduce the number of osteoclasts [12–14]. The effect of StR in healing fractures has been a subject of previous preclinical studies, often producing contradicting results [15, 16].

This study is aimed at investigating the effects of StR on fracture healing in normal mature female rabbits after two and four weeks of treatment. For this purpose, we used an ulnar fracture model and evaluated the healing process with a combination of histopathological, biomechanical, and radiological methods.

## 2. Materials and Methods

### 2.1. Study Design

The protocol for this experiment was approved by the Veterinary Authority, Directorate of Veterinary Medicine of the Prefecture of Evros, Greece (Licence Reference Number: T/2958/9-11-2010). Research was conducted in conformity with the National and European guidelines of laboratory animal care.

Forty skeletally mature New Zealand white female rabbits (age: 6-7 months; weight: 3.6–4.0 kg) were used for this study. Anesthesia induction was obtained with atropine sulfate at 0.04 mg/kg, ketamine hydrochloride (Imalgene®, Merial, Lyon-France) at 50 mg/kg, and xylazine (Rompun®, Bayer Corporation, Kansas-USA) at 5 mg/kg. All injections were given intramuscularly. If necessary, one-half of the induction dose was given intramuscularly to maintain anesthesia during surgery. After the rabbits were anaesthetized, a posterior longitudinal incision was made aseptically over the left ulna, the subcutaneous tissues were dissected, and the muscles were retracted exposing the middiaphysis of the underlying ulna. The periosteum on the osteotomy site was not elevated from the bone. An oscillating saw was used to create a transverse (width 1–2 mm) osteotomy at the middiaphysis of the ulna (45 mm distal to the olecranon process) on all animals. The osteotomies were not stabilized internally or externally. The subcutaneous tissues were closed primarily using a 3-0 PDS suture in a simple continuous pattern, taking care to bury all knots. The skin was closed in the same way. Full-weight bearing was allowed from the postoperative day 1 to the end of the study. After surgery, the animals were at random divided as follows: the study group consisted of 20 rabbits in which strontium ranelate (Protelos®, Les Laboratoires Servier, France) was administered at a dose of 600 mg/kg/day orally, for a period of two and four weeks. The control group consisted of 20 rabbits in which no StR was administered. Ten rabbits from each group were randomly euthanatized at 2 weeks and the remaining ten at 4 weeks after surgery using 5 mL of thiopental sodium (Pentothal®, Hospira UK Ltd, Warwickshire, UK) intravenous euthanasia solution and 10 mL potassium chloride endocardially. The bony specimens were harvested after removing muscles and soft tissues. The osteotomized forearms underwent radiographic examination. All osteotomized forearms were prepared for histopathological examination, while both intact and osteotomized bones underwent biomechanical testing. Samples for histopathological evaluation were stored in 10% neutral buffered formalin whereas ulnae for biomechanical examination were frozen at −20°C.

### 2.2. Radiography

Serial lateral and anteroposterior radiographs were taken postoperatively using a Siemens X-ray machine (Siemens, Erlangen, Germany). Callus maturity was evaluated at the second and fourth weeks. Union was considered complete when two cortices were bridging the site of the osteotomy, incomplete when only one cortex was bridging the osteotomy site, and nonunion when no bridging the osteotomy was present [17].

### 2.3. Biomechanical Testing

**Figure 1:**
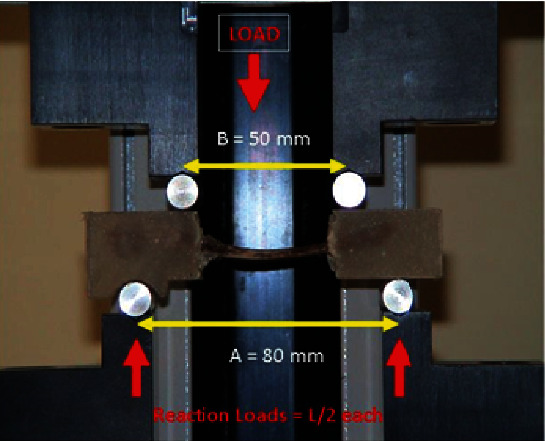
Mechanical testing machine for 4-point bending test.

A four-point bending test was performed at 2 and 4 weeks after study initiation. For both study groups, osteotomized and intact forearms were tested separately for each animal. A four-point bending test was carried out using a mechanical testing machine equipped with Load Cell ILC 2500 N (MECMESIN, UK: S/N: 03-1016-08; max travel = 500 mm; max load = 2500 N) [18]. Ten aluminum moulds, for accurate and reproducible specimen fixation, equipped with miniature setting screws for specimen holding were used. These moulds allow acrylic resin fixation of proximal and distal ulnar ends, ensuring a final specimen configuration compatible to the four-point bending setup. Specimens were first aligned and stabilized within the mould, so that the midpoint of the ulnar diaphysis (meant to be the host callus centerline) was centrally located. Subsequently, using soft modeling putty, the middle diaphyseal portion was carefully isolated from the proximal and distal bone compartments, where acrylic resin was poured for fixation. The moulds were specially designed and manufactured for this study, to ensure final specimen configuration compatible to the four-point bending setup of the laboratory. The mechanical testing machine is presented in Figure 1. The benefit of utilizing a four-point bending test is that the middle diaphyseal bone length is only subjected to a constant pure bending moment and is completely free of any shear contact that may unpredictably challenge or disturb the diaphyseal callus (as in the case of a three-point bending).

### 2.4. Histopathology

**Figure 2:**
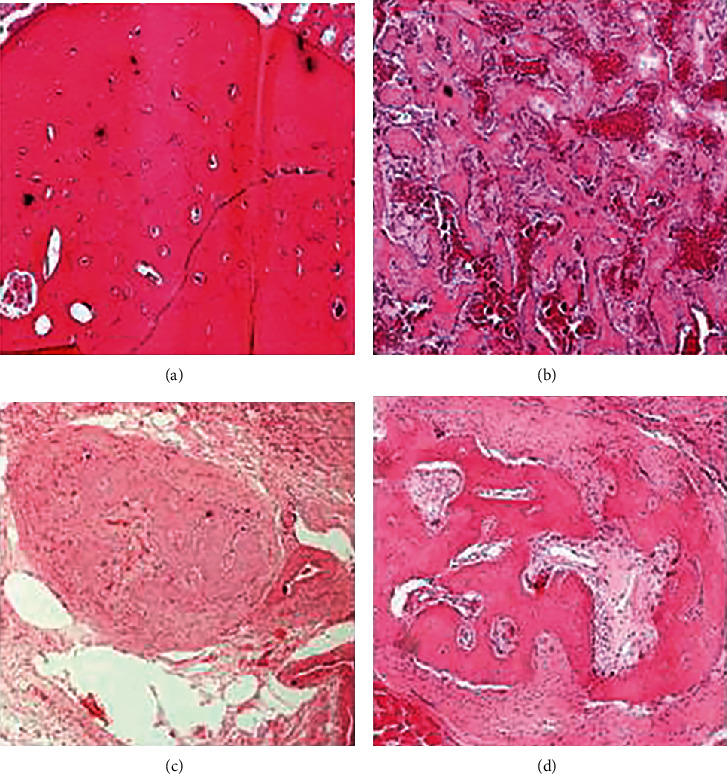
Total vessel number at 2 weeks in control and StR group (a, b) and New trabecular bone at 4 weeks in control and StR group (c, d). (a) Total vessel number in control group (magnification ×10, hematoxylin-eosin). (b) Total vessel number in StR group (magnification ×10, hematoxylin-eosin). (c) New trabecular bone in control group (magnification ×10, hematoxylin-eosin). (d) New trabecular bone in StR group (magnification ×10, hematoxylin-eosin). At 2 weeks, a significant increase of total vessel number was observed in the StR treatment group. At 4 weeks, a significant increase of new trabecular bone was observed after StR treatment.

Bones were fixed in 10% formalin solution and decalcified for two weeks. Five transverse consecutive sections from the callus area were taken and processed routinely to paraffin wax. Transverse sections were chosen for achieving the best measurement of the callus diameter. They were subsequently cut into 3 *μ*m sections and stained with hematoxylin and eosin. Tissues were examined and photographed under a light microscope (Nikon, Japan, model Eclipce E400). As previously suggested by Gerstenfeld et al. [19], the variables evaluated were as follows: (a) the callus diameter (mean value, in two orthogonal planes); (b) the total callus area (mean value for measurements of the total callus area); (c) the cartilaginous, fibrous, and osseous callus area, expressed as the percent of the total callus volume; (d) the number of osteoclasts per unit area of callus (mean value for all section measurements); (e) the number of osteoblasts per unit area of new trabecular bone (mean value for measurements made at three high power fields, for each section); and (f) the number of vessels per callus area (mean value for all section measurements) (Figure 2).

### 2.5. Statistical Analysis

Data are expressed as mean ± standard deviation (S.D.) or median (in case of violation of normality) for continuous variables and as percentages for categorical data. The Kolmogorov-Smirnov test was utilized for normality analysis of the parameters. The analysis of histomorphometric markers was performed using a two-way ANOVA model without repeated measurements, with the factors “intervention” (control vs. drug) and “time” (2 weeks vs. 4 weeks), to evaluate mean differences between groups. We performed multiple comparison tests adjusted by Bonferroni corrections.

The comparison of variables between groups at each leg (with and without osteotomy) was performed using the independent samples *t*-test or the Mann-Whitney test in case of violation of normality. The paired samples *t*-test was used for the comparison of the leg with and without osteotomy for each group. We calculated the new variables with using the formula osteotomy/without osteotomy ×100 in order to adjust our results to the normal leg. Comparison of the percentage ratio between the two groups was analyzed using the independent samples *t*-test or Mann-Whitney test in case of violation of normality. The analysis of radiological markers was performed using the Fisher exact test. All tests are two-sided, and statistical significance was set at *p* < 0.05. All analyses were carried out using the statistical package SPSS v.16.00 (Statistical Package for the Social Sciences, SPSS Inc., Chicago, III., USA).

## 3. Results

All animals completed the observation/treatment periods, and no deaths occurred prior to the designated euthanasia period of 2–4 weeks. During the entire study period, no wound infections were noted.

### 3.1. Histopathology Results

The treatment group presented statistically significant higher values of callus diameter (*p* < 0.001), total callus area (*p* < 0.001), percentage of fibrous tissue (*p* < 0.001), vessels/mm^2^ (*p* < 0.05), total vessel number (*p* < 0.05), and lower values for osteoclast number/mm^2^ (*p* < 0.05) compared with the control group at 2 weeks. Additionally, the treatment group presented statistically significant higher values of the percentage of new trabecular bone (*p* < 0.05), vessels/mm^2^ (*p* < 0.05), osteoclast number/mm^2^ (*p* < 0.05), and lower values for callus diameter (*p* < 0.05) and total callus area (*p* < 0.05) compared to the control group at 4 weeks. StR seems to reduce the number of osteoclasts during the early phase of fracture healing, while it increases both the diameter and callus area, as well as the percentage of fibrous tissue formation. The increase of vascularization was also significant compared with the control group. Therefore, early treatment with StR may beneficially affect fracture healing when compared with controls. In addition, as the healing progresses at 4 weeks, StR positively affects new trabecular bone formation and simultaneously decreases the callus size, thus promoting the physiological process of secondary fracture healing. Also, there was a statistically significant increase of callus diameter (*p* < 0.05), total callus area (*p* < 0.001), percentage of osseous tissue (*p* < 0.05), and total vessel number (*p* < 0.05) and decrease of percentage of cartilage (*p* < 0.05) and osteoclast number/mm^2^ (*p* < 0.05) in the specimen at 4 weeks compared with those at 2 weeks for the control group. Moreover, there was a statistically significant increase of the percentage of osseous tissue (*p* < 0.001), the percentage of new trabecular bone (*p* < 0.05), and the osteoclast number/mm^2^ (*p* < 0.05) in specimens at 4 weeks compared with those at 2 weeks and a decrease of callus diameter (*p* < 0.001), percentage of fibrous tissue (*p* < 0.001), and total osteoblast number/HPF (*p* < 0.05) in specimens at 4 weeks compared to those at 2 weeks for the treatment group. The histopathological results are presented in Table 1.

### 3.2. Biomechanical Results

At 2 weeks, biomechanical analysis was not feasible because all osteotomized samples failed immediately after load was applied. At 4 weeks, in the treatment group, force applied (*p* = 0.003), energy at failure (*p* = 0.004), and load at failure (*p* = 0.003) were all statistically significantly higher in the forearm specimens with osteotomized ulnae compared to those without. No significant differences were observed in the same parameters regarding the forearm specimens with and without osteotomized ulnae in the control group.

Moreover, no differences were observed between the control and treatment groups for all the above parameters in relation to forearm specimens with and without osteotomized ulnae except energy at failure for forearm specimen without osteotomized ulnae (*p* = 0.016).

With regard to the percentage ratio between the osteotomized and intact forearms, the treatment group presented statistically significantly higher values of force applied (133.6 ± 23.16 vs. 100.6 ± 21.22, *p* = 0.010), energy at failure (167.9 ± 49.0 vs. 112.9 ± 38.4, *p* = 0.026), and load at failure (133.6 ± 23.2 vs. 102.8 ± 17.8, *p* = 0.010) than the control group. The biomechanical results are presented in Table 2.

### 3.3. Radiological Results

**Figure 3:**
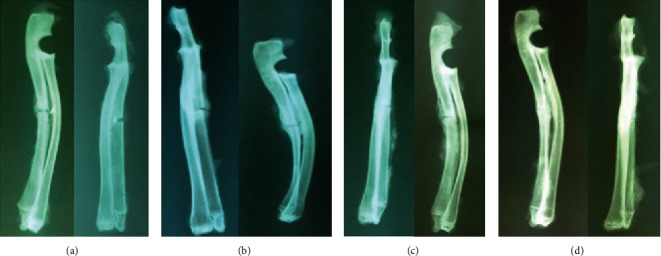
Fracture healing in control group at (a) 2 and (b) 4 weeks and StR group at (c) 2 and (d) 4 weeks.

No statistically significant differences were observed between the groups regarding radiologically observed bone union at 2 weeks (12.5% vs. 50%, *p* = 0.152). However, a greater statistical possibility of radiological bone union was demonstrated for animals receiving StR at 4 weeks compared with controls (62.5% vs. 26.7%, *p* = 0.045). Animals treated with StR presented a four-fold higher possibility of successful bone union compared with controls at 4 weeks after therapy (OR = 4.58; 95% CI: 1.03-15.06) (Figure 3).

## 4. Discussion

The effect of StR on fracture healing has been the subject of numerous studies. In the current study, evidence from histopathological, radiological, and biomechanical evaluations suggests that treatment with StR positively contributes to fracture healing.

Histopathological findings at 2 and 4 weeks demonstrate a beneficial effect of StR in fracture healing. In particular, at 2 weeks, an increase in callus diameter, total callus area, fibrous tissue formation, and vessel density and a decrease in osteoclast number/mm^2^ were observed in the study group receiving StR compared with the control group. At 4 weeks, a progressive reduction of the callus diameter, fibrous tissue, and osteoblast number/HPF, as well as an increase in bony tissue, such as osseous tissue and new trabecular bone, was observed in the study group compared to the study group at 2 weeks. These results suggest an enhancement of callus formation and maturity through the effects of StR on osteoblast/osteoclast function. These findings are in line with those found by Ammann et al. [14].

Biomechanical testing, which was performed using a four-point bending test, at 4 weeks revealed that all fractured ulnae broke away from the callus area, suggesting a beneficial effect of StR therapy on callus formation and resistance. Additionally, in the study group, the osteotomized forearms exhibited better mechanical properties compared with their nonosteotomized counterparts. This finding suggests that the effects of StR vary in healthy and fractured bone tissue. In the latter, StR seems to enhance the biomechanical variables of the callous compared to those of the intact bone. These results are in contrast to those of Bruel et al. [20] and Vegger et al. [21], who failed to show any biomechanical benefits of StR therapy. In particular, in the study of Bruel et al. [20], a three-point bending test showed that all fractured tibiae broke at the healing fracture site. These results support the superiority of utilizing a four-point bending test. This test has the benefit that only the middle diaphyseal bone length is subjected to a constant pure bending moment and is completely free of any shear contact that may unpredictably challenge and disturb diaphyseal callus (as in the case of a three-point bending). The four-point bending test was thought to better replicate the in vivo loading [22].

In the present study, radiological findings suggest an increased probability of callus formation, as evidenced by radiologic examination, in the group of animals receiving StR compared with controls in contrast to the study of Cebesoy et al. [23], where no such difference was noted.

Increasing evidence from clinical and experimental studies supports the beneficial effects of StR in enhancing fracture healing. In vitro studies demonstrated that StR reduces bone resorption [24, 25] and enhances bone formation [26]. In particular StR decreases the activity of osteoclasts and stimulates osteoblast multiplication and collagen synthesis [26]. In vivo studies also showed that StR inhibited bone resorption [27] and induced bone formation, as evidenced by histomorphometric investigation in rodents [28–30]. Similarly, Buehler et al. [12] found that treatment with StR decreased the histomorphometrical indices of bone resorption (osteoclast surface and number) and maintained bone formation in the alveolar bone in monkeys. Long-term treatment with StR increased bone mechanical properties by producing dose-dependent increases of bone strength and bone mass and improved bone microarchitecture, as assessed by increases of trabecular and cortical bone volumes and trabecular number and thickness [14]. In addition, treatment with StR prevented bone loss and microarchitecture degradation and improved intrinsic bone quality and endochondral ossification [31, 32]. Similar results were observed by Delannoy et al. [13] and Marie [33]. Moreover, results from preclinical studies demonstrated that StR therapy improves fracture healing by increasing callus formation, callus maturity, and mineralization, as well as by increasing callus volume and ameliorating biomechanical properties and bone quality [32, 34–38]. If only the parameter “energy at failure” is considered, it may not be safe to reach a safe conclusion regarding a negative effect of StR on healthy bone metabolism. One could hypothesize that StR may alter the crystalline structure of healthy bones, since an interchange, even though limited, occurs between StR and calcium with a maximum exchange of 1 molecule of StR to 10 molecules of calcium, when the former is administered in high dosages [39]. However, further research is needed in order to evaluate the effect of StR on healthy, nonosteotomized bones.

StR presents different activities, in terms of bone formation and healing procedure, in the normal mature bone compared with the fractured bone. In the study by Lavet et al. [40], proximal tibia bone defects were created in healthy rats and the animals were treated with StR for a period of 4, 8, or 12 weeks. The results showed that in the healing zone, treatment enhanced bone formation and decreased bone resorption, thus improving the healing process of both the cortical and trabecular compartments, with no deleterious effects on the newly formed bone. However, in the metaphyseal compartment, StR only decreased bone resorption without affecting bone formation.

It is well-known that metabolic conditions in osteoporotic bones differ from those in healthy bones. In osteoporosis, the rate of bone remodeling is augmented, causing increased bone resorption and decreased new bone formation. It is important to mention that in 2014, the European Medicines Agency's Pharmacovigilance Risk Assessment Committee (PRAC) has recommended that the medications Protelos and Osseor (strontium ranelate) should no longer be used to treat osteoporosis. The reason for this was a negative effect-benefit ratio of strontium ranelate, because these drugs show serious side effects. However, our study is aimed at evaluating the effects of short-term treatment with strontium ranelate on fracture healing in an animal model without osteoporosis. A recent study by Leiblein et al. [41] performed in healthy rats with femur fracture demonstrated that strontium ranelate presented a similar effect to parathormone (PTH) regarding new bone formation but shows low values for mineralization and biomechanical strength. In the current literature, data on the effect of strontium ranelate on fracture healing in humans is scarce; therefore, conclusions should be extrapolated with caution [34, 36, 42, 43], and obviously more research is needed.

Consequently, in the early phase of fracture healing, inhibition of extreme bone resorption and enhancement of bone formation from StR treatment may contribute to increased callus volume and bone density [32]. In the majority of animal studies, there is evidence of a beneficial effect of StR on fracture healing [32, 35, 37, 44, 45]. However, in some studies, treatment with StR failed to create the appropriate conditions in order to achieve effective fracture healing [46].

Additionally, the procedure of fracture healing is significantly different when fracture ends are in close contact (gap of 1–2 mm) compared with fractures with a larger gap. Evidence regarding the effect of StR in the healing of fractures presenting a considerable gap has been conflicting. Results from the studies of Zacchetti et al. [38] and Lavet et al. [40] reveal that treatment with StR in fractures with a standardized drill hole defect (2.5 mm wide and 2 mm deep) leads to an acceleration of fracture healing, filling of bone defect, and increased bone volume. Conversely, Ibrahim et al. [16] suggested that the effects of StR treatment in rabbits with a 5 mm bone fracture gap begun slowly and probably caused a delay in the acute stage of fracture healing. Similar results were also observed by Vegger et al. [21]. In our study, we used healthy, adult, female rabbits and we induced an ulnar fracture, which is 45 mm below the olecranon, without creating a gap. The integrity of the periosteum was preserved. The periosteum represents an important source of pluripotent cells and provides approximately 1/3 of the cortical blood flow, making it indispensable for the bone healing process [47]. Additionally, the total absence of the periosteum and lesions of the intramedullary vascular network has been associated with fracture nonunion [47].

The strength of our study is that the effect of StR on fracture healing was evaluated using a combination of 3 separate methods: radiological, histopathological, and biomechanical. To date, this is the only study evaluating the effect of StR treatment on fracture healing using a 4-point bending test, since this type of biomechanical analysis better approaches the physiologic loading conditions, in comparison to a three-point bending test. No internal or external fixation was used for fracture stabilization. Fixation of the osteotomy was not required because of the particularly strong interosseous membrane of the rabbit's forearms [48]. Additionally, the intact radius acted as an immobilization splint on the osteotomy [49, 50]. Moreover, during the daily observation, it was noted that the osteotomized legs were loaded equally to the healthy legs.

In contrast with other studies [13, 14, 20], in our study, StR was administered in each rabbit orally through a syringe and not mixed in the food; thus, a precise daily dose of the drug, as defined by the study protocol, was guaranteed. The treatment dose used was proportional to that used in humans for the treatment of osteoporosis [32].

Our study presents certain limitations. The administered StR dose (600 mg/kg/day) should create mean serum StR concentrations that correspond to the clinical dose of 2 g/day, but the actual serum StR concentration was not determined in our study. In addition, the observation time of 2 and 4 weeks did not provide adequate data regarding the early stages of fracture healing. Finally, there was no additional evaluation of the callus size using microcomputed tomography or nanoindentation.

## 5. Conclusion

In conclusion, the present study demonstrates a potentially beneficial effect of StR in fracture healing. These results create new alternatives for the use of StR as a pharmacologic agent with a potential role on bone defect repair. Further studies evaluating its possible role in promoting fracture healing are necessary.

## Figures and Tables

**Table 1:** Comparison of histopathological results between groups receiving StR and controls at 2 and 4 weeks after study initiation.

	Control 2 w	Control 4 w	StR 2 w	StR 4 w	*p*_between 4 groups_
Callus diameter (mm)	8.81 ± 0.75	11.06 ± 2.56^∗^	11.55 ± 1.34^∗∗^	8.38 ± 1.16^$##^	<0.0001
Total callus area (mm^2^)	46.13 ± 15.58	104.88 ± 26.25^∗∗^	88.10 ± 26.94^∗∗^	72.25 ± 16.60^∗^^$^	<0.0001
% cartilage	24.38 ± 18.01	5.00 ± 7.07^∗^	13.00 ± 9.19	5.00 ± 10.04^∗^	0.007
% fibrous tissue	23.75 ± 7.91	21.88 ± 6.51	38.00 ± 6.32^∗∗^^$$^	19.38 ± 9.43^##^	<0.0001
% osseous tissue	48.13 ± 18.31	66.88 ± 10.67^∗^	45.00 ± 9.43^$^	71.88 ± 12.52^∗∗^^##^	<0.0001
% new trabecular bone	60.00 ± 15.20	70.00 ± 13.10	73.00 ± 13.37	85.00 ± 5.35^∗∗^^#$^	0.004
Total osteoclast number	43.63 ± 16.28	63.00 ± 26.39	52.60 ± 24.69	59.38 ± 36.98	0.502
Osteoclast number/mm^2^	0.99 ± 0.39	0.59 ± 0.19^∗^	0.61 ± 0.21^∗^	0.86 ± 0.45^#$^	0.041
Osteoblast number/HPF	26.38 ± 7.7	19.38 ± 11.20	35.30 ± 12.38^$^	18.50 ± 7.76^#^	0.005
Total vessel number	18.38 ± 12.34	46, 50 ± 34.84^∗^	77.40 ± 50.24^∗^^$^	59.88 ± 22.54^∗∗^	0.001
Vessels/mm^2^	0.38 ± 0.16	0.46 ± 0.31	0.74 ± 0.33^∗^	0.93 ± 0.48^∗^^$^	0.012

We used the two-way ANOVA model without repeated measurements, with factors the “intervention” (control vs. drug) and “time” (2 weeks vs. 4 weeks). Multiple comparisons were performed using the Bonferroni test. Abbreviations: HPF: high-power field. ^∗^*p* < 0.05 vs. control 2 weeks and ^∗∗^*p* < 0.001 vs. control 2 w. ^$^*p* < 0.05 vs. control 4 weeks and ^$$^*p* < 0.001 vs. control 4 w. ^#^*p* < 0.05 vs. StR 2 weeks and ^##^*p* < 0.001 vs. StR 2 w.

**Table 2:** Comparison of biomechanical results between groups receiving StR and controls in forearms with and without osteotomy at 4 weeks after study initiation.

		Without osteotomy	Osteotomy	*p*_within group_	% (O/WO)	*p*_%(O/WO)_
Force	Control	666.58 ± 162.83	663.30 ± 205.68	NS	100.6 ± 21.22	0.010
	StR	552.13 ± 82.64	734.91 ± 154.71	0.003	133.6 ± 23.16	
	*p*_between group_	NS	NS			

Energy	Control	873.25 ± 150.62	998.38 ± 432.19	NS	112.9 ± 38.4	0.026
	StR	666.25 ± 150.61	1081.00 ± 271.16	0.004	167.9 ± 49.0	
	*p*_between group_	0.016	NS			

Moment	Control	5.00 ± 1.22	5.13 ± 1.55	NS	102.8 ± 17.8	0.010
	StR	4.14 ± 0.62	5.51 ± 1.16	0.003	133.6 ± 23.2	
	*p*_between group_	NS	NS			

The comparison of variables between groups at each leg (with and without osteotomy) was performed using the independent samples *t*-test or the Mann-Whitney test in case of violation of normality. The paired samples *t*-test was used for the comparison of the leg with and without osteotomy for each group. We calculated the variable %(O/WO) = osteotomy/without osteotomy × 100 in order to adjust our results to the normal leg. Comparison of the percentage ratio between the two groups was analyzed using the independent samples *t*-test or Mann-Whitney test in case of violation of normality. Abbreviations: O: osteotomy; WO: without osteotomy; NS: nonsignificant.

## Data Availability

The data used to support the findings of this study are available from the first author, Ourania I. Koukou, rkoukou@ http://yahoo.com, upon request.

## References

[1] Bonafede M., Espindle D., Bower A. G. (2012). The direct and indirect costs of long bone fractures in a working age us population. *Journal of Medical Economics*.

[2] Einhorn T. A., Gerstenfeld L. C. (2015). Fracture healing: mechanisms and interventions. *Nature Reviews Rheumatology*.

[3] Shantz J. S., Marcucio R., Kim H. T., Miclau T., Court-Brown C. M., Heckman J. D., McQueen M. M., Ricci W. M., Tornetta P. (2015). Failure of fracture healing-etiologies and overview of treatment strategies. *In Rockwood and Green's fractures in adults*.

[4] Einhorn T. A. (1995). Enhancement of fracture-healing. *The Journal of Bone and Joint Surgery. American Volume*.

[5] Antonova E., Le T. K., Burge R., Mershon J. (2013). Tibia shaft fractures: costly burden of nonunions. *BMC Musculoskeletal Disorders*.

[6] Kates S. L., Ackert-Bicknell C. L. (2016). How do bisphosphonates affect fracture healing?. *Injury*.

[7] Marie P. J., Felsenberg D., Brandi M. L. (2011). How strontium ranelate, via opposite effects on bone resorption and formation, prevents osteoporosis. *Osteoporosis International*.

[8] Reginster J. Y., Kaufman J. M., Goemaere S. (2012). Maintenance of antifracture efficacy over 10 years with strontium ranelate in postmenopausal osteoporosis. *Osteoporosis International*.

[9] Bonnelye E., Chabadel A., Saltel F., Jurdic P. (2008). Dual effect of strontium ranelate: stimulation of osteoblast differentiation and inhibition of osteoclast formation and resorption in vitro. *Bone*.

[10] Atkins G. J., Welldon K. J., Halbout P., Findlay D. M. (2009). Strontium ranelate treatment of human primary osteoblasts promotes an osteocyte-like phenotype while eliciting an osteoprotegerin response. *Osteoporosis International*.

[11] Brennan T. C., Rybchyn M. S., Green W., Atwa S., Conigrave A. D., Mason R. S. (2009). Osteoblasts play key roles in the mechanisms of action of strontium ranelate. *British Journal of Pharmacology*.

[12] Buehler J., Chappuis P., Saffar J. L., Tsouderos Y., Vignery A. (2001). Strontium ranelate inhibits bone resorption while maintaining bone formation in alveolar bone in monkeys (Macaca fascicularis). *Bone*.

[13] Delannoy P., Bazot D., Marie P. J. (2002). Long-term treatment with strontium ranelate increases vertebral bone mass without deleterious effect in mice. *Metabolism*.

[14] Ammann P., Shen V., Robin B., Mauras Y., Bonjour J. P., Rizzoli R. (2004). Strontium ranelate improves bone resistance by increasing bone mass and improving architecture in intact female rats. *Journal of Bone and Mineral Research*.

[15] Fonseca J. E., Brandi M. L. (2010). Mechanism of action of strontium ranelate: what are the facts?. *Clinical Cases in Mineral and Bone Metabolism*.

[16] Ibrahim M. R. M., Singh S., Merican A. M. (2016). The effect of strontium ranelate on the healing of a fractured ulna with bone gap in rabbit. *BMC Veterinary Research*.

[17] Marsh D. (1998). Concepts of Fracture Union, Delayed Union, and Nonunion. *Clinical Orthopaedics and Related Research*.

[18] Turner C. H., Burr D. B. (1993). Basic biomechanical measurements of bone: a tutorial. *Bone*.

[19] Gerstenfeld L. C., Wronski T. J., Hollinger J. O., Einhorn T. A. (2005). Application of Histomorphometric Methods to the Study of Bone Repair. *Journal of Bone and Mineral Research*.

[20] Brüel A., Vegger J. B., Raffalt A. C., Andersen J. E. T., Thomsen J. S. (2013). PTH (1–34), but not strontium ranelate counteract loss of trabecular thickness and bone strength in disuse osteopenic rats. *Bone*.

[21] Vegger J. B., Brüel A., Sorensen T. G., Thomsen J. S. (2016). Systemic treatment with strontium ranelate does not influence the healing of femoral mid-shaft defects in rats. *Calcified Tissue International*.

[22] Fischer K. J. (1999). Low-profile versus conventional metacarpal plating systems: a comparison of construct stiffness and strength. *The Journal of Hand Surgery (A)*.

[23] Cebesoy O., Tutar E., Kose K. C., Baltaci Y., Bagci C. (2007). Effect of strontium ranelate on fracture healing in rat tibia. *Joint, Bone, Spine*.

[24] Baron R., Tsouderos Y. (2002). In vitro effects of S12911–2 on osteoclast function and bone marrow macrophage differentiation. *European Journal of Pharmacology*.

[25] Takahashi N., Sasaki T., Tsouderos Y., Suda T. (2003). S12911–2 inhibits osteoclastic bone resorption in vitro. *Journal of Bone and Mineral Research*.

[26] Canalis E., Hott M., Deloffre P., Tsouderos Y., Marie P. J. (1996). The divalent strontium salt S12911 enhances bone cell replication and bone formation in vitro. *Bone*.

[27] Marie P. J., Hott M. (1986). Short-term effects of fluoride and strontium on bone formation and resorption in the mouse. *Metabolism*.

[28] Marie P. J., Garba M. T., Hott M., Miravet L. (1985). Effect of low doses of stable strontium on bone metabolism in rats. *Mineral and Electrolyte Metabolism*.

[29] Grynpas M. D., Marie P. J. (1990). Effects of low doses of strontium on bone quality and quantity in rats. *Bone*.

[30] Arlot M. E., Roux J. P., Boivin G. (1995). Effect of strontium salt (S12911) on both tibial metaphysis and epiphysis in normal growing rats. *Journal of Bone and Mineral Research*.

[31] Bain S. D., Jerome C., Shen V., Dupin-Roger I., Ammann P. (2009). Strontium ranelate improves bone strength in ovariectomized rat by positively influencing bone resistance determinants. *Osteoporosis International*.

[32] Li Y. F., Luo E., Feng G., Zhu S. S., Li J. H., Hu J. (2010). Systemic treatment with strontium ranelate promotes tibial fracture healing in ovariectomized rats. *Osteoporosis International*.

[33] Marie P. J. (2005). Strontium ranelate: a novel mode of action optimizing bone formation and resorption. *Osteoporosis International*.

[34] Alegre D. N., Ribeiro C., Sousa C., Correia J., Silva L., de Almeida L. (2012). Possible benefits of strontium ranelate in complicated long bone fractures. *Rheumatology International*.

[35] Habermann B., Kafchitsas K., Olender G., Augat P., Kurth A. (2010). Strontium ranelate enhances callus strength more than PTH 1-34 in an osteoporotic rat model of fracture healing. *Calcified Tissue International*.

[36] Tarantino U., Celi M., Saturnino L., Scialdoni A., Cerocchi I. (2010). Strontium ranelate and bone healing: report of two cases. *Clinical Cases Mineral Bone Metabolism*.

[37] Ozturan K. E., Demir B., Yucel I., Cakıcı H., Yilmaz F., Haberal A. (2011). Effect of strontium ranelate on fracture healing in the osteoporotic rats. *Journal of Orthopaedic Research*.

[38] Zacchetti G., Dayer R., Rizzoli R., Ammann P. (2014). Systemic treatment with strontium ranelate accelerates the filling of a bone defect and improves the material level properties of the healing bone. *BioMed Research International*.

[39] Boivin G., Deloffre P., Perrat B. (1996). Strontium distribution and interactions with bone mineral in monkey iliac bone after strontium salt (S12911) administration. *Journal of Bone and Mineral Research*.

[40] Lavet C., Mabilleau G., Chappard D., Rizzoli R., Ammann P. (2017). Strontium ranelate stimulates trabecular bone formation in a rat tibial bone defect healing process. *Osteoporosis International*.

[41] Leiblein M., Henrich D., Fervers F., Kontradowitz K., Marzi I., Seebach C. (2020). Do antiosteoporotic drugs improve bone regeneration in vivo?. *European Journal of Trauma and Emergency Surgery*.

[42] Negri A. L., Spivacow F. R. (2012). Healing of subtrochanteric atypical fractures after strontium ranelate treatment. *Clinical Cases Mineral Bone Metabolism*.

[43] Scaglione M., Fabbri L., Casella F., Guido G. (2016). Strontium ranelate as an adjuvant for fracture healing: clinical, radiological, and ultrasound findings in a randomized controlled study on wrist fractures. *Osteoporosis International*.

[44] Komrakova M., Weidemann A., Dullin C. (2015). The impact of strontium ranelate on metaphyseal bone healing in ovariectomized rats. *Calcified Tissue International*.

[45] da Rosa J. A., de São Paulo U., Brazil K. K. S. (2016). Strontium ranelate effect on the repair of bone defects and molecular components of the cortical bone of rats. *Brazilian Dental Journal*.

[46] Fuchs R. K., Allen M. R., Condon K. W. (2008). Strontium ranelate does not stimulate bone formation in ovariectomized rats. *Osteoporosis International*.

[47] Neagu T. P., Tiglis M., Cocolos I., Jecan C. R. (2016). The relationship between periosteum and fracture healing. *Romanian Journal of Morphology and Embryology*.

[48] Bostrom M., Lane J. M., Tomin E. (1996). Use of bone morphogenetic protein-2 in the rabbit ulnar non-union model. *Clinical Orthopaedics and Related Research*.

[49] Waters R. V., Gamradt S. C., Asnis P. (2009). Systemic corticosteroids inhibit bone healing in a rabbit ulnar osteotomy model. *Acta Orthopaedica Scandinavica*.

[50] Karachalios T., Boursinos L., Poultsides L., Khaldi L., Malizos K. N. (2007). The effects of the short-term administration of low therapeutic doses of anti-COX-2 agents on the healing of fractures. *The Journal of Bone and Joint Surgery*.

